# A high ratio of linoleic acid (n-6 PUFA) to alpha-linolenic acid (n-3 PUFA) adversely affects early stage of human neuronal differentiation and electrophysiological activity of glutamatergic neurons *in vitro*

**DOI:** 10.3389/fcell.2023.1166808

**Published:** 2023-05-15

**Authors:** Karolina Dec, Mouhamed Alsaqati, Joanne Morgan, Sumukh Deshpande, Jamie Wood, Jeremy Hall, Adrian J. Harwood

**Affiliations:** ^1^ Neuroscience and Mental Health Innovation Institute, School of Medicine, Cardiff University, Cardiff, Wales, United Kingdom; ^2^ Division of Psychological Medicine and Clinical Neurosciences, School of Medicine, Cardiff University, Cardiff, Wales, United Kingdom; ^3^ School of Pharmacy, Newcastle University, Newcastle Upon Tyne, England, United Kingdom; ^4^ School of Medicine, Cardiff University, Cardiff, Wales, United Kingdom; ^5^ School of Biosciences, Cardiff University, Cardiff, Wales, United Kingdom

**Keywords:** polyunsaturated fatty acids, neurodevelopment, human induced pluripotent stem cells, linoleic acid, alpha-linolenic acid, GABA signaling

## Abstract

**Introduction:** There is a growing interest in the possibility of dietary supplementation with polyunsaturated fatty acids (PUFAs) for treatment and prevention of neurodevelopmental and neuropsychiatric disorders. Studies have suggested that of the two important classes of polyunsaturated fatty acids, omega-6 (n-6) and omega-3 (n-3), n-3 polyunsaturated fatty acids support brain development and function, and when used as a dietary supplement may have beneficial effects for maintenance of a healthy brain. However, to date epidemiological studies and clinical trials on children and adults have been inconclusive regarding treatment length, dosage and use of specific n-3 polyunsaturated fatty acids. The aim of this study is to generate a simplified *in vitro* cell-based model system to test how different n-6 to n-3 polyunsaturated fatty acids ratios affect human-derived neurons activity as a cellular correlate for brain function and to probe the mechanism of their action.

**Methods:** All experiments were performed by use of human induced pluripotent stem cells (iPSCs). In this study, we examined the effect of different ratios of linoleic acid (n-6) to alpha-linolenic acid in cell growth medium on induced pluripotent stem cell proliferation, generation of neuronal precursors and electrophysiology of cortical glutamatergic neurons by multielectrode array (MEA) analysis.

**Results:** This study shows that at a n-6:n-3 ratio of 5:1 polyunsaturated fatty acids induce stem cell proliferation, generating a large increase in number of cells after 72 h treatment; suppress generation of neuronal progenitor cells, as measured by decreased expression of FOXG1 and Nestin in neuronal precursor cells (NPC) after 20 days of development; and disrupt neuronal activity *in vitro*, increasing spontaneous neuronal firing, reducing synchronized bursting receptor subunits. We observed no significant differences for neuronal precursor cells treated with ratios 1:3 and 3:1, in comparison to 1:1 control ratio, but higher ratios of n-6 to n-3 polyunsaturated fatty acids adversely affect early stages of neuronal differentiation. Moreover, a 5:1 ratio in cortical glutamatergic neurons induce expression of GABA receptors which may explain the observed abnormal electrophysiological activity.

## Introduction

Nutrients in our diet play an important role in maintaining human body homeostasis. It is widely described that diet poor in certain vitamins, elements, or essential fatty acids (EFAs) may lead to abnormal physiology of many organs. Two major classes of polyunsaturated fatty acids (PUFAs), omega-6 (n-6) and omega-3 (n-3) play a key role in neurodevelopment and brain function (Balanzá-Martínez et al., 2011; Bazinet et al., 2014; Lacombe et al., 2018). Although metabolism and consumption of PUFAs is very high in many tissues, including the brain, they cannot be synthesized in mammals due to a lack of relevant enzymes (Hejr et al., 2017) and must be delivered in the diet as n-6 linoleic acid (LA; 18:2n-6) and n-3 alpha-linolenic acid (ALA; 18:3n-3) (Kang et al., 2014; Knobloch, 2017). LA and ALA are then converted, mainly liver, into long-chain PUFAs (LC-PUFAs) of n-6 and n-3 families, especially arachidonic acid (AA; 20:4n-6), docosahexaenoic acid (DHA, 22:6n-3) and eicosapentaenoic acid (EPA; 20:5n-3). PUFAs themselves play a role in regulating membranes fluidity (they are incorporated into cell membranes), but under certain conditions, the LC-PUFAs are further released and metabolised into biologically active mediators (oxylipins). These include prostaglandins (PG), leukotrienes (LT), lipoxins (LX), thromboxanes (TX), resolvins (Rv), maresins and protectins (Lauritzen et al., 2016). Oxylipins act as signalling molecules to indirectly regulate cell survival, proliferation, inflammation, gene expression and synaptogenesis (Chapkin et al., 2009; Bazinet et al., 2014; Kang et al., 2014). n-6 PUFA metabolites often mediate negative or proinflammatory signals, whereas n-3 PUFAs are anti-inflammatory and have beneficial effects on brain development and function (Hejr et al., 2017). Studies suggest that the most relevant PUFA in the brain is DHA (n-3), which has beneficial effects on brain development and function (Lacombe et al., 2018). It can enhance the generation of neuronal progenitor cells, and induce synapse formation, neurite outgrowth and the number of neurite branches (Katakura et al., 2009; Katakura et al., 2013; Yamamoto et al., 2021). DHA incorporation in foetal tissues depends on maternal dietary supply. During the third trimester of pregnancy DHA accumulates to higher levels in the brain than in other parts of the body, while AA (n-6) accumulation in CNS is in line with that observed in other tissues (Cetin et al., 2009; Lauritzen et al., 2016).

In the last few decades, dietary patterns of developing countries have changed due to increased consumption of high energy, but poor in nutritional-value foods due to higher intake of processed meals. This has led to increased consumption of red meat, sugars and alcohol and decreased consumption of fruits, vegetables, whole grains, fish, and nuts (Knobloch, 2017; Dagnino-Subiabre, 2021). This so called Western or American Diet has higher intake of n-6 dietary lipids, increasing the ratio of n-6 to n-3 PUFAs, departing from a healthy value of below 3:1 and rising to between 10:1 to 50:1. The Western Diet is also known to be proinflammatory due to higher synthesis of proinflammatory n-6 lipid derivatives. Neuropsychiatric disorders present an additional burden for global health, with rising numbers of individuals diagnosed with developmental and mood disorders. These patients often experience a reduced quality of life and increased mortality; hence it is important to develop appropriate prevention and treatment strategies (Wainberg et al., 2017). Epidemiological studies showed that the occurrence of mood disorders is correlated with low plasma levels of n-3 PUFAs (Dagnino-Subiabre, 2021), and research is needed to evaluate the effect of n-3 fatty acids as a therapy for psychiatric disorders in children and adults. To date, clinical trials have been inconclusive, and we lack understanding of the required dosage, PUFA specificity and treatment duration. Further investigations are required to provide reliable guidelines for the use of PUFAs as a supportive supplementation in psychiatric disorders treatment and in prevention of neurodevelopmental disorders (Bernal et al., 2018; Bozzatello et al., 2021; Wolters et al., 2021).

The aim of this study is to generate a simplified human *in vitro* cell-based model to test the effect of n-6:n-3 PUFA ratios on neuronal differentiation and neuronal network activity. Neuronal development is tightly regulated by many transcription factors and signalling molecules, and PUFAs may influence these processes (Kang et al., 2014; Hettige et al., 2019). It is well known that lipids are required for brain development, however, there is a gap between basic knowledge of the molecular pathways that are regulated by lipids and data obtained from population-based studies. Understanding the role of PUFAs and their metabolites in neuronal cell fate regulation at a molecular level is crucial for developing guidance on possible use of lipids in prevention and therapy of neuropsychiatric disorders.

## Material and methods

### hiPSCs culture and neuronal differentiation

Experiments were performed using a hiPSCs control cell line IBJ4 (derived from fibroblast line ATCC; CRL-2522). hiPSCs were cultured in Essential 8 Medium (Gibco) on matrigel (Corning). Control IBJ4 were differentiated into cortical glutamatergic neurons using slightly modified dual SMAD protocol (Chambers et al., 2009; Shi et al., 2012). For neurodifferentiation, cells were passaged on 12-well plates on growth factor reduced matrigel (Corning) and cultured in Essential 8 Medium (Gibco) until approximately 90% confluency, when they were switched to N2B27 medium (DMEM/F12 (Gibco), Neurobasal (Gibco), 0.3xB27-RA (without retinoic acid) (0.3 dilution from ×50 concentrated stock; Gibco), 0.6xN2 (0.6 dilution from ×100 concentrated stock; Gibco), 1xPSG (×1 dilution from ×100 concentrated stock; Gibco), 0.1 mM *ß*-mercaptoethanol (Gibco), 10 nM SB431542 (Sigma-Aldrich) and 100 nM LDN193189 (Sigma-Aldrich), designated as Day 0, (D0). At Day 9 (D9) cells were passaged on fibronectin (Sigma Aldrich) coated plates and at Day 10 (D10) changed to medium without SB and LDN. At Day 20 (D20) the cells reached NPC stage and neuronal rosettes were visible in the culture, they were passaged on PDL/laminin (Sigma Aldrich) coated plates. Further passages on PDL/laminin were performed only if necessary (usually another one at D30). At D26 media was switched to N2B27+ RA (with retinoic acid), and neurons maintained on laminin coated plates until D60. Standard protocol for generation cortical neurons uses B27 supplement which contains LA (n-6) and ALA (n-3) at ratio 1:1. In this study, we aim to investigate the effect of different PUFA ratios on neuronal development and function. To play with the lipid ratio we generated our in-house replacement for B27, NS21 supplement (Chen et al., 2008). We tested the effect of PUFA ratios at three stages of neuronal differentiation which were stem cell stage (D0), NPCs stage (D20) and neuronal stage (D60). Cells were supplemented with Essential 8 Medium (Gibco) containing four different ratios of LA (n-6) to ALA (n-3) PUFAs (1:1; 1:3; 3:1 and 5:1) to investigate their influence on cell proliferation rate. For neurodifferentiation, cells were cultured in tested PUFA ratios from D0 until D20, NPC stage. To investigate, the effects of PUFA ratios on neuronal activity, we differentiated neurons to D40 using standard N2B27 medium and then switched medium for N2NS21+ RA (with different PUFA ratios) for the following 20 days. Samples from above-described experiments were collected at D0, D20 and D60 for analysis.

### Cell number analysis using cellinsight CX7 high-content screening platform

Control cells (IBJ4) were dissociated with Accutase (Thermo Fisher) to obtain single cell solution and plated with the density 5,000 per well on 24-well plates. The number of cells per 1 mL of cell suspension was calculated with automatic Millipore Scepter Cell Counter (Merck). Stem cells were treated with different ratios of PUFA for 24, 48, and 72 h. At each timepoint plates were fixed with 3.7% paraformaldehyde (PFA) and stained with DAPI (Thermo Fisher), nuclei marker. The number of cells for each condition and each timepoint was calculated using CellInsight CX7 High-Content Screening Platform (Thermo Fisher). We then compared the increase in cell number in time for IBJ4 treated with PUFAs to establish if PUFA ratio influence cell proliferation.

### RNA extraction and quantitative PCR

Cell pellets were collected at D0, D20 and D60. On the day of collection, culture media was aspirated, cells were washed once with DPBS (Gibco) and incubated for 3 min at 37°C with Versene (Gibco). After 3 min, Versene was removed, and pellets were collected and stored at −20°C for further analysis. RNA was extracted using RNAeasy MiniKit (Qiagen), and cDNA was generated using 0,75 ug of RNA with a high-capacity cDNA reverse transcription kit (Applied Biosystems). For qRT-PCR, all samples were analysed in technical triplicates, using qPCRBIO SyGreen Blue Mix Hi-ROX (PCR Biosystems). The mRNA abundance was calculated using 2^−ΔΔCT^ and data was normalised to *b-actin*.

### The analysis of changes in transcriptome using next-generation sequencing (NGS)

For the bulk RNA sequencing (RNAseq) analysis control IBJ4 were treated with PUFAs as previously described and the pellets were collected at D20 of differentiation (n = 3 each condition). RNA was extracted using RNAeasy Plus MiniKit (Qiagen). All samples were quality checked using fragment analyser (Agilent) and quantified using Qubit™ RNA High Sensitivity (HS) Assay (Thermo Fisher Scientific). Libraries were generated using KAPA mRNA HyperPrep Kit (Roche) and pooled in an equimolar fashion, then sequenced on an Illumina NovaSeq 6,000, using a S2 flow cell with 100 base paired-end sequencing. Differential Expressed Gene (DEG) analysis was performed using the DESeq2 package in R. The enrichment analysis was performed on all differentially expressed genes (DEGs) using the Enrichr package.

### Electrophysiology analysis with use of Multielectrode arrays (MEA)

All experiments were performed using CytoView MEA 24-well plates (M384-tMEA-24W, 4X4 electrode grid), pre-treated with 0.01% polyethylenimine (Sigma) for 1 h at 37°C. Day 40 neurons were plated at high density (5,000 cells/µL) with 10 μg/mL laminin and 0.5 mL of N2B27 medium added after 1 h, which was replaced after 24 h, 72 h, then with N2NS21 medium supplemented with one of four n-6 to n-3 PUFA ratios 1:1, 1:3, 3:1 and 5:1. MEA cultures were maintained in these PUFA ratios with medium replacement every 3–4 days. Electrophysiological activity was recorded every 10 days of incubation using a Maestro Pro MEA system with a Maestro 768-channel amplifier and AxIS 1.5.2 software (Axion Biosystems Inc., Atlanta, GA). Channels were sampled simultaneously with a gain of ×1,000 and a sampling rate of 12.5 kHz/channel. During the recording, the temperature was maintained constant at 37°C.

A Butterworth band-pass filter (with a high-pass cut-off of 200 Hz and low-pass cut-off of 3000 Hz) was applied along with a variable threshold spike detector set at 5.5 × standard deviation on each channel. Offline analysis was achieved with custom scripts written in MATLAB (available on request). Briefly, spikes were detected from filtered data using an automatic threshold-based method set at −5.5 × σ, where σ is an estimate of the noise of each electrode based upon the median absolute deviation 1. Spike timestamps were analysed to provide statistics on the general excitability of cultures. Network activity was illustrated by creating array–wide spike detection rate (ASDR) plots with a bin width of 200 ms. Synchronized burst number, duration and interval for each culture were derived with the Axion built-in neural metric analysis tool employing the envelope algorithm. This algorithm defines a synchronized burst (SB) by identifying times when the histogram exceeds a threshold of 1.25 standard deviation above or below the mean with a minimum of 200 ms between SBs and at least 35% of electrodes included.

### Statistical analysis

Statistical analysis was performed using Prism 9.0 (GraphPad). The difference between PUFA ratios treatments was tested using one-way or two-way ANOVA, and *p* < 0.05 was considered as statistically significant. The data was checked for outliers using the ROUT method (Q = 10%), and all outliers were removed from the dataset. All graphs show mean values and the standard error of the mean (SEM).

## Results

### n-6 PUFAs increase proliferation rate in iPSC culture *in vitro*

**FIGURE 1:**
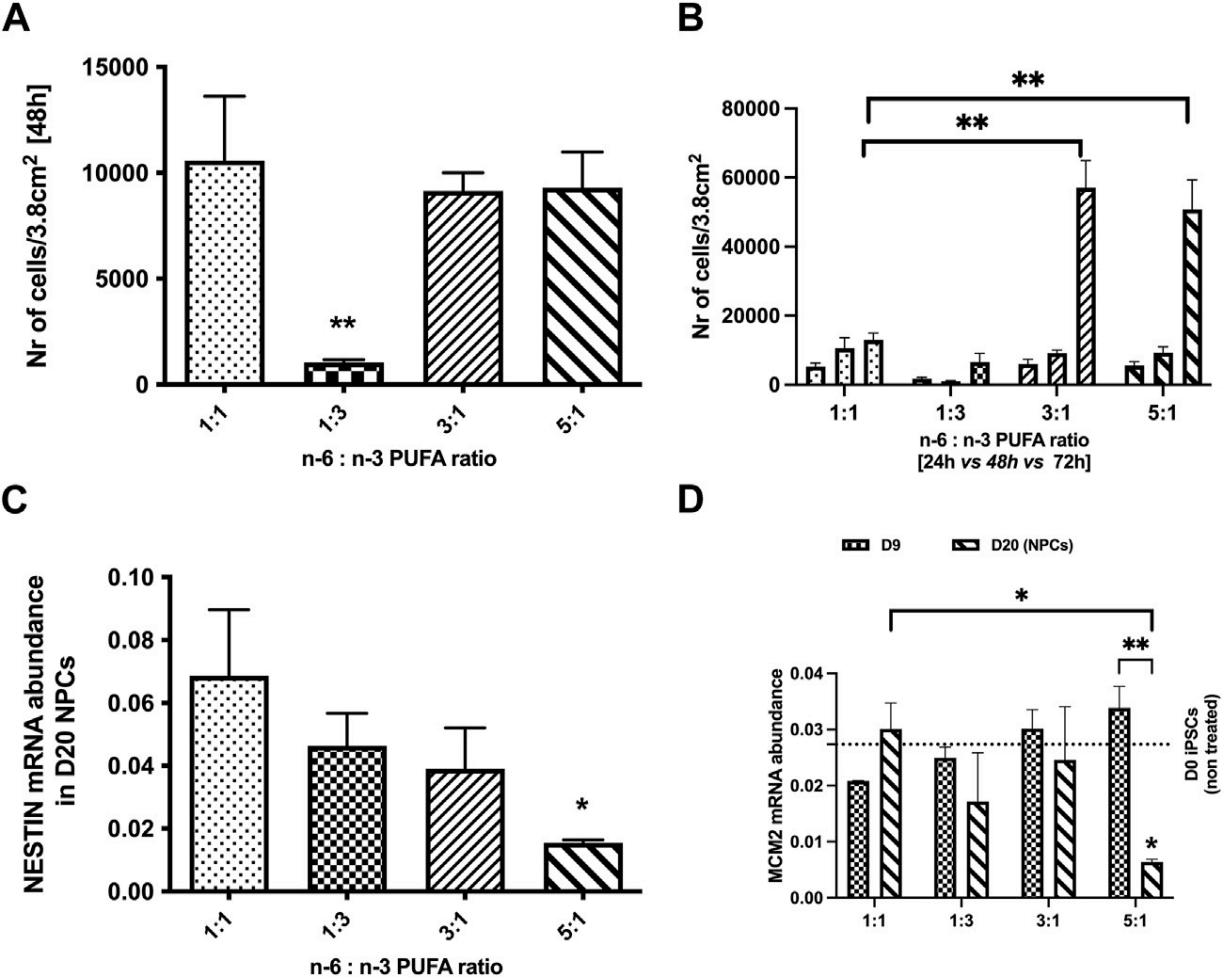
Effects of n-6:n-3 PUFA ratios on iPSC proliferation rate and proliferation markers expression in neuronal progenitor cells (NPCs). IPSCs were plated on 24-well plates with the density 5,000 cells per well and treated with culture medium containing four different PUFA ratios: 1:1, 1:3, 3:1, and 5:1. **(A)** The number of cells measured after 48 h with lipids; **(B)** the difference in the increase in number of cells for each condition between 24, 48, and 72 h time points. The number of cells at each timepoint for three tested PUFA ratios (1:3, 3:1, 5:1) was compared to the control 1:1 ratio. Each bar represents mean ± SEM. *p < 0.05, **p < 0.01, following one-way ANOVA, n = 6. **(C, D)** The expression of proliferation markers; **(C)** Nestin and **(D)** Minichromosome Maintenance Complex Component 2 (MCM2) in D20 NPCs and D9 for MCM2 **(D)** in 1:3, 3:1, 5:1 PUFA ratios(Each bar represents mean ± SEM. *p < 0.05, **p < 0.01, following two-way ANOVA for MCM2 and one-way ANOVA for Nestin, n = 6 (two independent differentiations).

We first examined whether PUFA ratios influence hiPSC proliferation. Previous literature suggested that n-6 fatty acids alone regulate cell metabolism and affect cell growth, proliferation and apoptosis (Kang et al., 2014). Hence, we tested whether various n-6 to n-3 PUFA ratios can affect cell growth and the proliferation rate. IBJ4 hiPSC were dissociated to a single cell suspension and cultured at a density 5,000 cells per well in different ratios of PUFAs for 24, 48, and 72 h. Cell growth medium was changed every day for the wells treated for 48 and 72 h to ensure that iPSCs are maintained in a constant level of PUFAs. After 48 h a significant reduction in cell number per well was seen for a 1:3 PUFA ratio compared to control 1:1 PUFA ratio (*p* < 0.05, one-way ANOVA, Figure 1A.), and this difference persisted to 72 h. In contrast, a greater than 5-fold increase in cell number per well was observed at 72 h in cultures with 3:1 and 5:1 PUFA ratios containing more n-6 than n-3 PUFAs (mean = 8,838, 49,172 and 50,745 for 1:1, 3:1 and 5:1 ratios, respectively, *p* < 0.05, one-way ANOVA, Figure 1B.).

We further investigated the impact of PUFA ratios on NPCs proliferation (D20 of neurodevelopment). hiPSCs were differentiated in a range of PUFA ratios to D20 and analysed for gene expression of the cell proliferation genes Nestin (neuroepithelial stem cell protein) and Minichromosome Maintenance Complex Component 2 (MCM2) (Bernal et al., 2018). No significant differences in Nestin and MCM2 expression were observed between NPCs treated with 1:3 and 3:1 PUFA ratios when compared to the control 1:1 ratio (*p* < 0.05, one-way ANOVA). In contrast to the increased of cell number seen for pluripotent hiPSC cultures, a high PUFA ratio (5:1 ratio) significantly decreased expression of both proliferation genes at D20 NPC stage (*p* < 0.05, one-way ANOVA, Figures 1C, D). Interestingly, no difference of MCM2 expression was observed at D9 indicating that the negative effects of high PUFA ratios emerges after the initial stages on neurodifferentiation.

### High n-6 PUFA ratios have a detrimental effect on early-stage *in vitro* neurodevelopment

**FIGURE 2:**
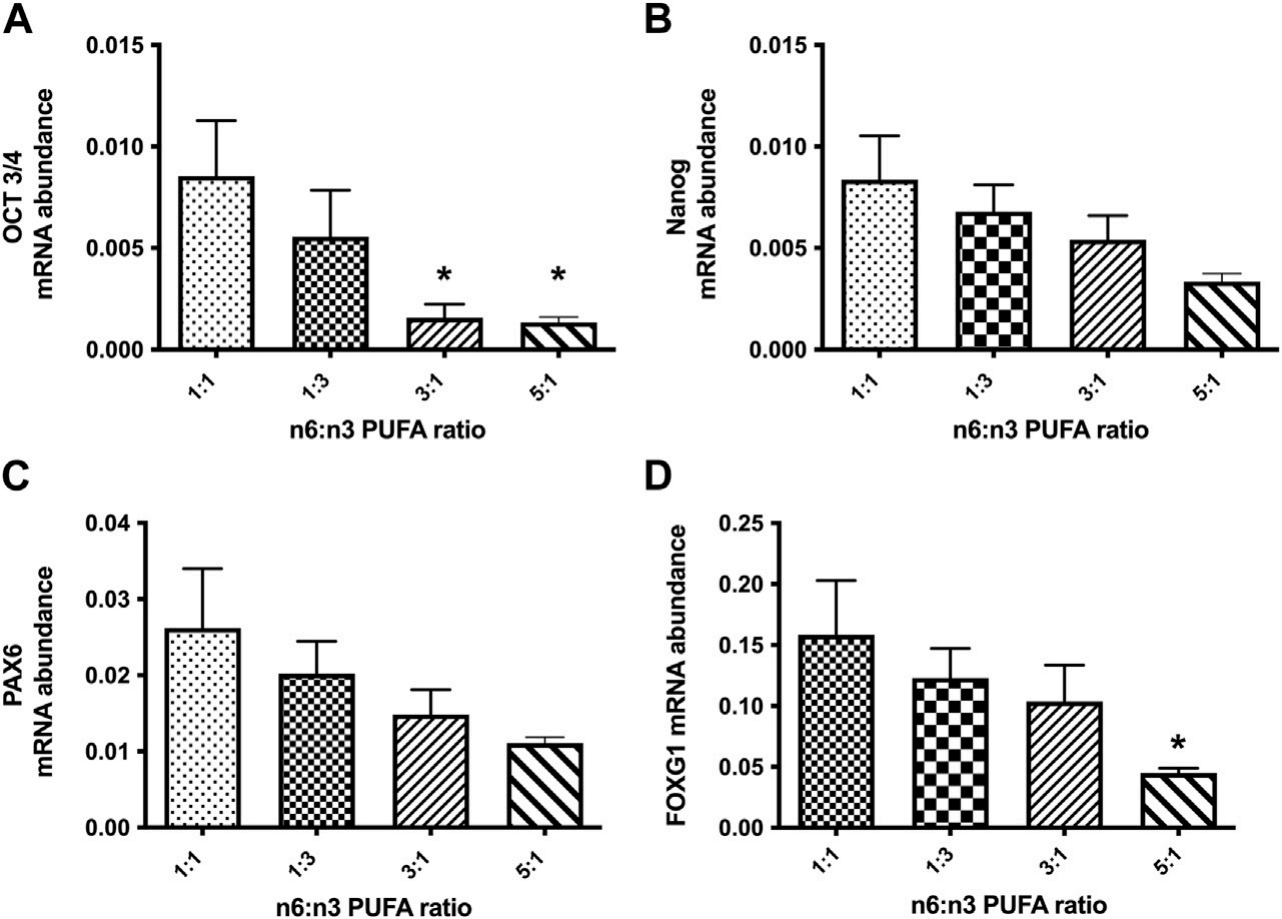
Relative expression analysis of stem cell markers **(A, B)**: (OCT-3/4, *Nanog*) and neuronal precursor markers **(C, D)**: (PAX6, FOXG1) in NPCs, following 20 days treatment with medium containing different ratios of n-6:n-3 PUFAs: 1:1(control), 1:3, 3:1 and 5:1. Each bar represents mean ± SEM. *p < 0.05, following one-way ANOVA, n = 6 (two independent differentiations).

Population based studies report that supplementation of n-3 LC-PUFAs during pregnancy is beneficial for developing brain and may also improve adult brain function (Bazinet et al., 2014). A key stage of neurodevelopment *in vitro* cultures is formation of NPC, where cell-fates are laid down to later determine neurons or glia cell subtypes. We examined the effect of PUFA ratio on developmentally regulated gene expression at the NPC stage by qRT-PCR. Expression of pluripotent stem cell markers OCT-3/4 (octamer-binding transcription factor 3/4, encoded by the *Pou5f1* gene) and Nanog (Rodda et al., 2005; Stefanovic et al., 2007)*.* Both 3:1 and 5:1 caused a significant decrease of OCT3/4 expression, although this was not strongly borne with *Nanog gene* expression (Figures 2A, B). We next examined expression of the NPC transcription factor PAX6 (paired-box transcription factor 6) and FOXG1 (forkhead box G1); PAX6 is expressed in early neurodifferentiation, whereas FOXG1 is induced during later stages of NPC differentiation. As for the pluripotent gene markers at the NPC stage we observed no statistically significant changes in expression of all tested markers between cells treated with 1:3 PUFA ratio and the control 1:1 PUFA ratio (Figures 2A–D; *p* < 0.05, one-way ANOVA), However, a significant decrease of FOXG1 was observed at the high 5:1 n-6 PUFA ratio (Figure 2D), but again not strongly bourns out for PAX6 (Fog 2C). Taken together these results indicate that high n-6:n-3 PUFA ratio has a profound effect on early stages of *in vitro* neurodifferentiation, however this is not a simple case of cells remaining in the pluripotent, proliferating state.

To probe the neurodevelopmental suppression further, we carried out an RNA-seq analysis of D20 NPCs (n = 3 for each PUFA ratio). For NPCs treated with PUFAs, ratio 1:3 compared to a 1:1 ratio we found no DEGs. In contrast increasing the n-6 PUFAs created 4,065 and 4,272 DEGs for 3:1 and 5:1 PUFA ratios respectively. A total of 3,694 DEGs were common to 3:1 and 5:1 PUFA ratios. Cells treated with 5:1 ratio showed the 573 DEGs, whilst ratio 3:1 showed unique 371 DEGs. These data indicate again that an increase in n-3 PUFAs (1:3) ratio has neither positive nor negative effects on early stages of *in vitro* neurodevelopment, whereas increasing n-6 PUFAs has a major developmental impact.

### n-3 PUFAs enhances and n-6 PUFAs suppresses neuronal network synchronisation

**FIGURE 3:**
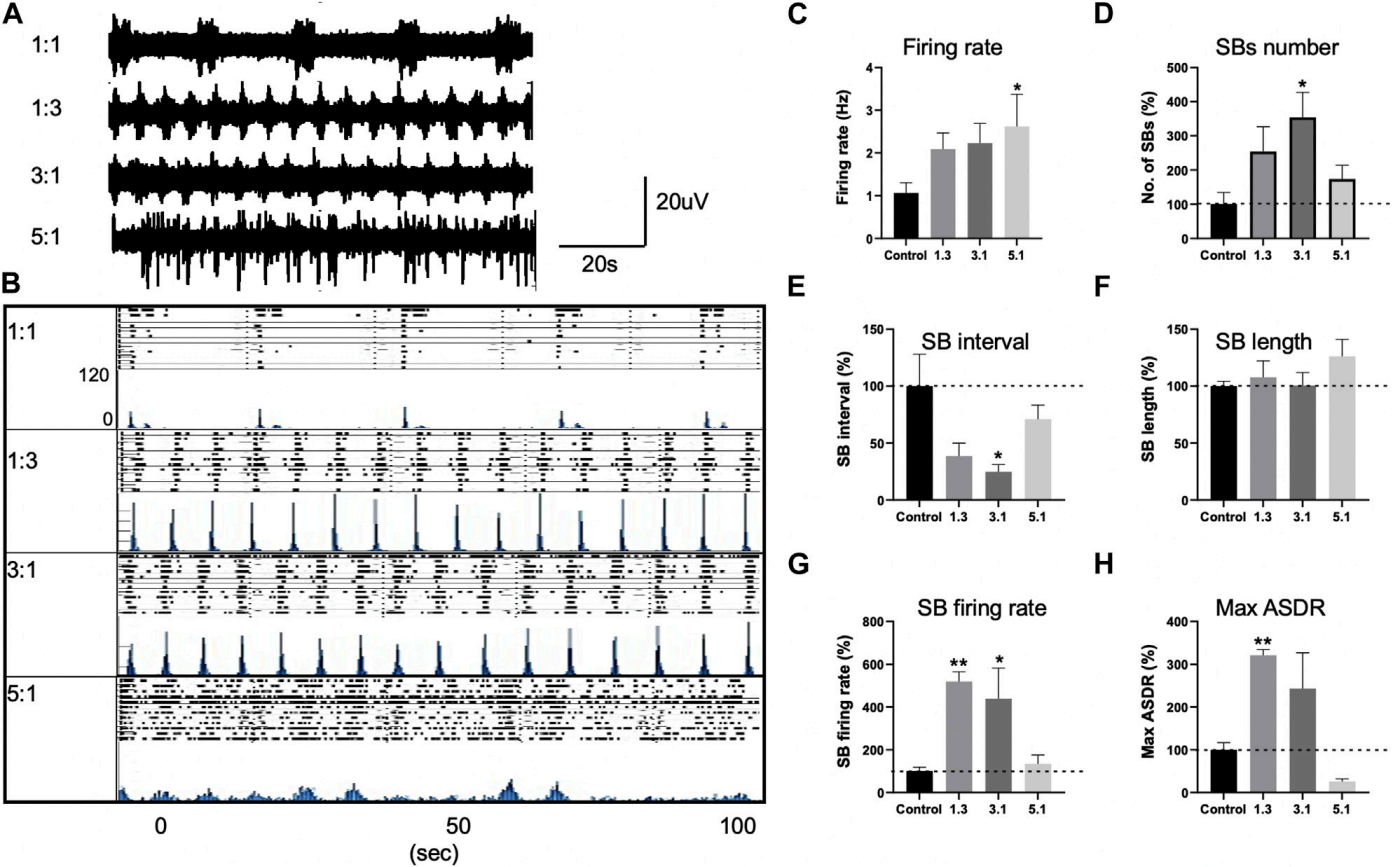
Changes in neuronal activity of control neurons treated with various PUFA ratios, 1:1, 1:3, 3:1 and 5:1 and recorded with multi electrode arrays (MEAs). **(A)** Representative voltage traces from three electrodes of the same MEA cultures treated with 1:1, 1:3, 3:1, and 5:1 PUFA ratios at 20DPP (DPP, days post plating). **(B)** Development of array–wide spike detection rate (ASDR) and raster plots for control 1:1 and 1:3, 3:1, 5:1 treated neurons. For each time point, upper panel shows raster plot and lower panel shows ASDR plot. Vertical scale bars = 120 spikes per 200 ms bin, following 100 s of recording. Raster plot of 100 s of recording demonstrating the synchronized burst (SB) duration and interval of neurons treated with various lipid ratios and generated by Axion built-in neural metric analysis tool. Basal excitability of control neurons treated with various lipid ratios showing mean firing rate **(C)**. Changes in synchronized burst (SB) frequency **(D)**, SB interval **(E)**, length **(F)**, the number of spikes in individual SBs **(G)** and maximum ASDR **(H)** in neurons treated with different lipid ratios. Values of panels **(D–H)** are shown as percentage of those seen in the control 1:1 ratio. All plots show means ± SEM. *p < 0.05, **p < 0.01 following one-way ANOVA. Number of cultures = 6–9.

To understand the consequence of higher n-6:n-3 PUFA ratio, we carried out a multielectrode array (MEA) analysis of D60 neurons treated with our four different ratios of PUFAs; 1:1, 1:3, 3:1 and 5:1 for 20 days prior to recording of neuronal activity and burst synchronicity. Spike detection from filtered raw voltage recordings were generated from each electrode via the threshold-based method of Quiroga and others and following quality control spikes were time-stamped for subsequent analysis (Quiroga et al., 2004). PUFA ratios of 1:3 and 3:1 had no effect on the spontaneous neuronal excitability with no significant difference in the mean firing rate compared to control (1:1) ratio cultures (Figures 3A–C). For example, mean firing rate was 1.063 ± 0.23 Hz, 2.087 ± 0.3 Hz, and 2.229 ± 0.46 Hz in the presence of 1:1, 1:3 and 3:1, respectively (*p* > 0.05, one-way ANOVA, Figure 3C). In contrast, a 5:1 n-6 PUFA ratio significantly increased the spontaneous firing rate of the neurons; the mean firing rate was 1.063 ± 0.23 Hz in the control cells while 2.61 ± 0.75 Hz in the presence of the lipid ratio 5:1 (*p* < 0.05, one-way ANOVA, Figure 3C). This indicates that whereas higher n-3 PUFA ratios had no significant impact on the intrinsic neuronal excitability, increasing n-6 PUFAs to 5:1 ratio elicits a measurable increased firing rate.

We then examined how PUFA ratios effect the neuronal network behavior. Following maturation, control (1:1) neuronal cultures developed a regular pattern of synchronized bursts (SBs), where burst firing is observed synchronously across all or many MEA electrodes, separated by intervals of similar length (Figures 3A, B), consistent previous observations (Plumbly et al., 2019; Alsaqati et al., 2020). In neurons treated with 1:3 or 3:1 PUFA ratios we observed the emergence of higher rates of synchronized bursting earlier than in control 1:1 ratio culture. These higher n-3 and intermediate n-6 PUFA ratios possessed significantly increased SB firing rate and maximum ASDR values (*p* > 0.01, one-way ANOVA, Figures 3G, H) for 1:3 PUFA ratios and threefold increase in SB frequency (*p* < 0.05, one-way ANOVA, Figure 3D). The increase in the SB frequency was accompanied by a significant decrease in SB interval (distance between the bursts; *p* < 0.05, one-way ANOVA, Figure 3E) for 3:1 PUFA ratio. In contrast, neurons treated with a higher 5:1 n-6 PUFA ratio exhibited no clear pattern of SBs, an indication of less synoptically connected cultures, (Figures 3A, B, D) (Plumbly et al., 2019). While the SB length, SB interval and SB firing rate did not significantly differ between the control and 5:1 treated neurons (*p* > 0.05, one-way ANOVA, Figures 3E, F, G), the maximum ASDR decreased by 75% but was not statistically significant (*p* > 0.05, one-way ANOVA, Figure 3H). Combined, these data indicate that incubation with higher n-3 and intermediate n-6 PUFA ratios enhanced neuronal connectivity, whereas neurons treated with a high, 5:1 PUFA ratio showed neuronal hyperexcitability, but with reduced network activity.

To investigate the underlying changes that drive altered network activity seen in a high PUFA ratio, we examined our RNAseq data for D20 NPCs. This showed strong similarities between 3:1 (subthreshold) ratio and 5:1 PUFA ratio. Interestingly, the gene cluster with the most significant enrichment for 3:1 and 5:1 PUFA ratio was GABA receptor family proteins (Table 1.).

**TABLE 1:** The most significantly differentially expressed genes (DEGs) related to GABA signalling in D20 neuronal progenitor cells (NPCs). Adjusted *p*-value cut off at *p* < 0.001; Log_2_FC cut off at ±1.5.

Gene	3:1 n-3:n6 PUFA ratio		5:1 n-3:n6 PUFA ratio	
	Log_2_FC	*p*-value	Log_2_FC	*p*-value
GABRA1	3.66	07.46E-15	4.09	1.87E-20
GABRA2	3.27	8.67E-128	3.56	1.67E-233
GABRB1	7.61	2.55E-12	7.48	4.26E-12
GABRB2	2.62	1.05E-56	2.86	8.83E-128

**FIGURE 4:**
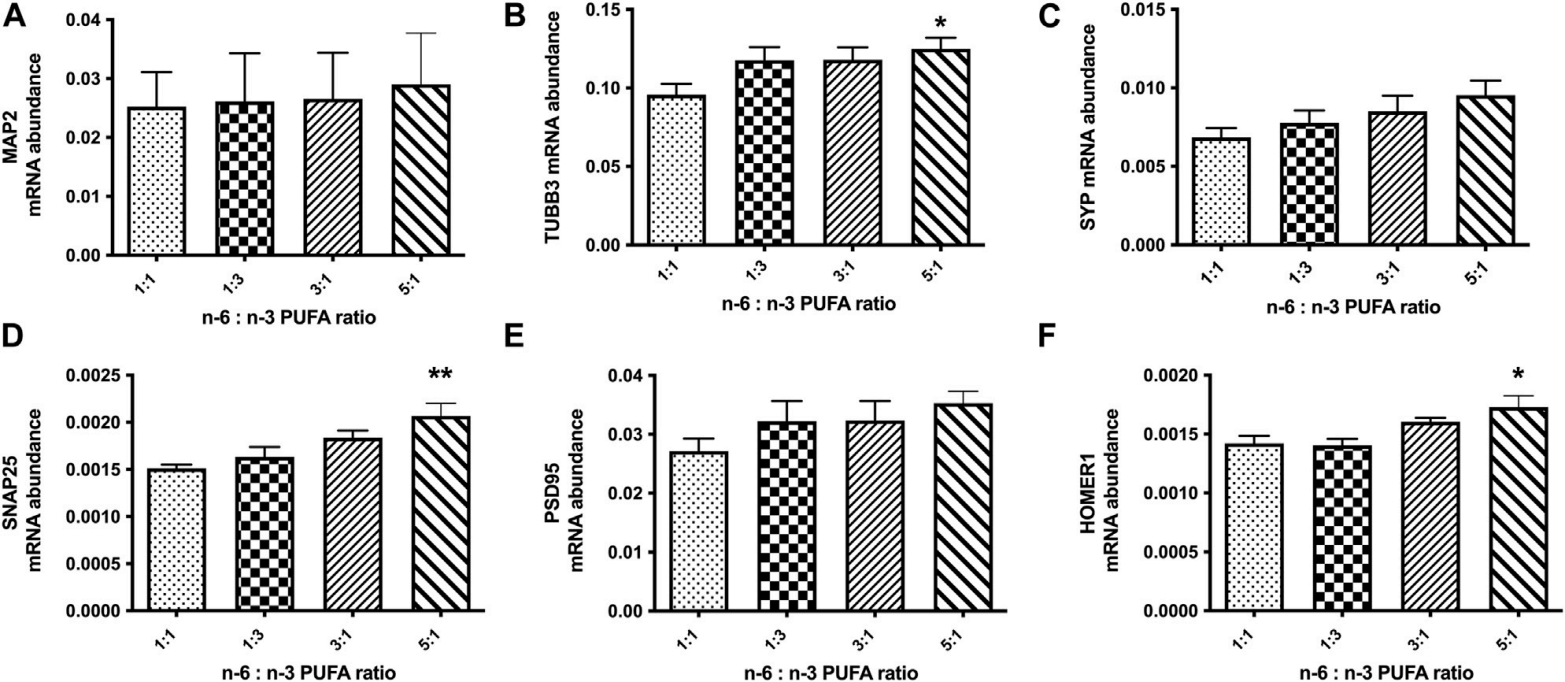
Relative expression analysis of neuronal marker genes **(A, B)**: (MAP2, TUBB3), pre-synaptic markers **(C, D)**: (SYP, SNAP25) and post-synaptic markers **(E, F)**: (PSD95, HOMER1) in D50 neurons, after 10 days treatment with medium containing different ratios of n-6:n-3 PUFA: 1:1(control), 1:3, 3:1 and 5:1. Each bar represents mean ± SEM. *p < 0.05, **p < 0.01, following one-way ANOVA, n = 6 (two independent differentiations).

This suggested that in high n-6 PUFA concentrations, NPC may follow an altered neurodevelopment trajectory to form neurons with an elevated sensitivity to GABA, ultimately leading to excitatory/inhibitory imbalance seen in our MEA cultures. We therefore used qRT-PCR to analyse gene expression in D60 neurons. Initially, we tested the mRNA expression of the neuronal marker genes microtubule associated protein 2 (MAP2), tubulin Beta 3 class III (TUBB3) and the synaptic marker genes synaptophysin (SYP), synaptosomal-associated protein 25 (SNAP25), postsynaptic density protein 95 (PSD95) and HOMER1 (Figures 4A–F). These are well-established markers, commonly used to monitor neuronal differentiation. The results of this experiment showed statistically significant increase in relative expression of TUBB3, SNAP25 and HOMER1 (Figures 4B, D, F; *p* < 0.05, one-way ANOVA) in cells cultured with medium containing 5:1 ratio of PUFAs when compared to control 1:1 ratio. This suggests a potential increase of synaptogenesis in high n-6 PUFA ratios.

**FIGURE 5:**
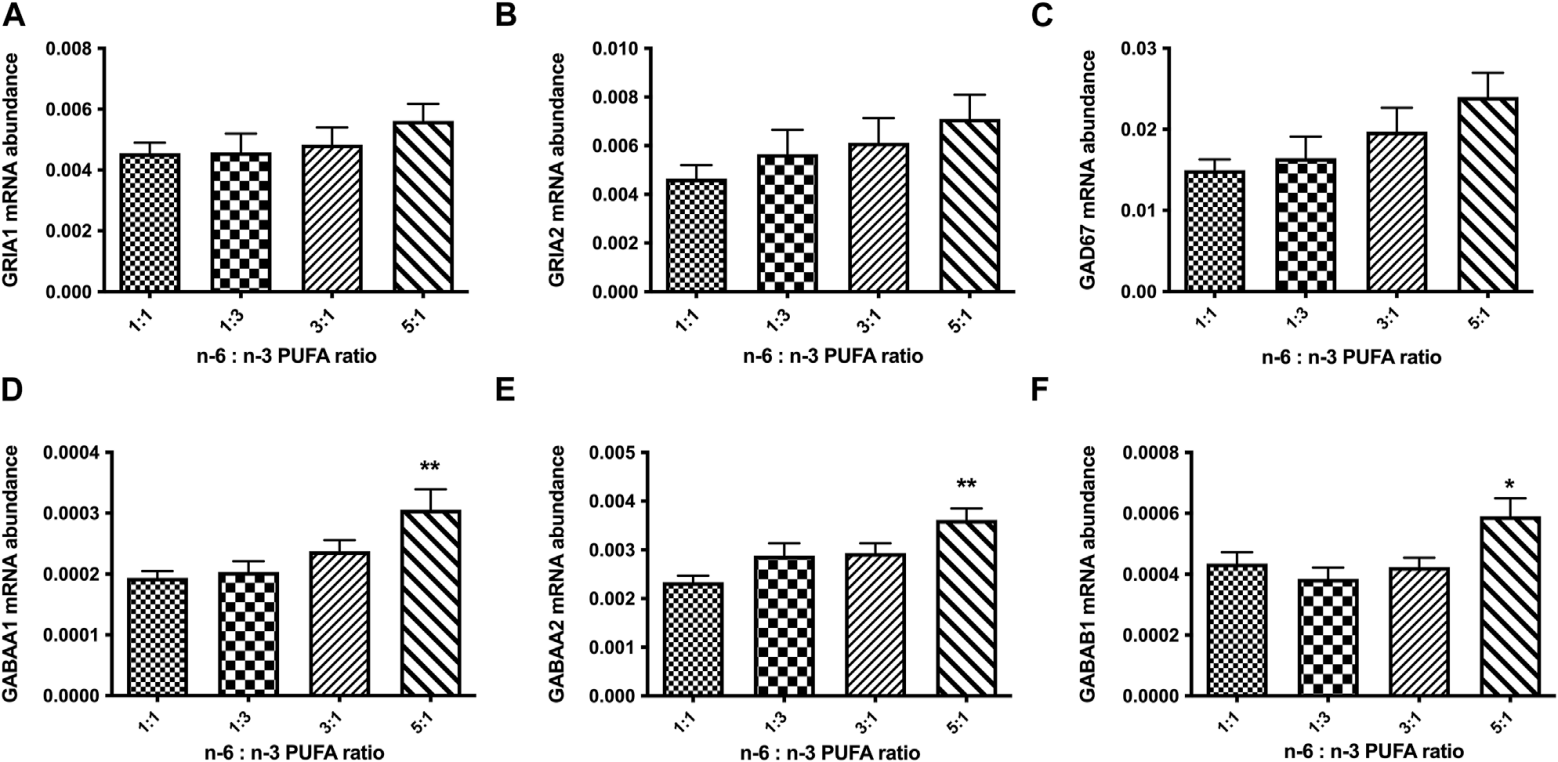
Relative expression analysis of glutamate **(A, B)**: (GRIA1, GRIA2), and GABA **(C–F)**: (GAD67, GABA_A_α1, GABA_A_α2, GABA_A_β1) signalling proteins in D50 neurons, after 10 days of treatment with medium containing different ratios of n-6:n-3 PUFA: 1:1(control), 1:3, 3:1 and 5:1. Each bar represents mean ± SEM. *p < 0.05, **p < 0.01, following one-way ANOVA, n = 6 (two independent differentiations).

We also tested the expression of glutamatergic synapse receptors, glutamate ionotropic receptor AMPA type subunit 1 (GRIA1) and glutamate ionotropic receptor AMPA type subunit 2 (GRIA2) and GABA signalling protein genes glutamate decarboxylase 67 (GAD67) and GABA receptor subunit genes for GABA_A_α_1_, GABA_A_α_2_, and GABA_A_β_1_ (Figures 5A–F). The results show no significant changes in relative expression of glutamatergic receptor genes for any tested ratio compared to control (Figures 5A, B; *p* < 0.05, one-way ANOVA). However, we observed statistically significant increase in expression GABA_A_ receptor subunits α_1_, α_2_, and β_1_ in cells treated with 5:1 ratio of PUFAs (Figures 5D–F; *p* < 0.05, one-way ANOVA). These data suggest that an increase in n-6:n-3 PUFA ratio may alter the balance in excitatory/inhibitory (E/I) signalling in neuronal culture *in vitro*.

## Discussion

Application of human iPSCs for *in vitro* disease modelling offers a feasible means of analysis for the effect of genetic variation, chemical compounds, or nutrients (such as PUFAs) on neuronal development (McNeill et al., 2020). Here we present a human *in vitro* cell model system for investigation of the neurocellular mechanisms of PUFA action on neurodevelopment and neuronal activity. Previous studies suggest that PUFA-derived signal mediators (oxylipins) may regulate cell survival and proliferation and influence cell fate. As a consequence, changes of the dietary n-6:n-3 PUFA ratio may have substantial impact on neurodevelopment (Hou et al., 2013; Kang et al., 2014). Most studies have focussed on testing either a single class of PUFAs (n-6 or n-3) or their derivatives, but not the ratio in a mixture of PUFA types. Here, we test the effects of the LA (n-6) and ALA (n-3) ratios on both stem cells proliferation and neuronal differentiation, with the aim of more closely matching the environmental exposure. We find that a high PUFA ratio (5:1) has a strong effect on both stem cell proliferation, neuronal differentiation and neuronal network activity.

We make a number of key observations. First, a high n-6 PUFA ratio enhances cell proliferation in pluripotent iPSC, but we find no evidence for increased proliferation once cells enter the neurodevelopmental pathway. In fact, development in a high n-6 PUFA ratio led to decreased expression of the two proliferation markers MCM2 and Nestin at the NPC stage compared to standard medium with a 1:1 n-6:n-3 PUFA ratio. Interestingly, no significant changes in MCM2 and Nestin expression were observed after 9 days of treatment with high n-6 PUFA ratio, suggesting that its negative effects arise only later in NPC. This stage-dependent switch in proliferative behaviour indicates that increased proliferation observed in pluripotent iPSC is not simply due to a failure to enter neurodevelopment.

The effects of n-6 PUFAs on pluripotent iPSC proliferation have also been seen for studies with n-6 PUFA metabolites PGE2 and Arachidonic acid (ARA) (Yun et al., 2009; Porter et al., 2013; Kang et al., 2014). PGE2 binds to its receptor EP1 and regulates the cell cycle via an EP1-dependent protein kinase C (PKC) and Phosphoinositide 3-kinase (PI3K)/Akt signalling pathways (Yun et al., 2009). Moreover, Liou and others reported that the same signalling pathways are responsible for cell resistance to apoptosis, raising the possibility that the increased in the number of cells after 72 h treatment with a high n-6 PUFAs ratio may be in part due to increased cell survival (Liou et al., 2007). Additional studies suggest that other PUFA derivatives (leukotrienes and thromboxanes) also influence cell proliferation and control cell cycle and metabolism (Wada et al., 2006; Yun et al., 2009).Taken together, these reports suggest that it may be the n-6 PUFA metabolites that maintain pluripotency, proliferation and cell survival.

Second, we also find that high n-6 PUFA ratio (5:1) negatively affect neuronal differentiation *in vitro*, decreasing expression of OCT-3/4 gene (Abu-Remaileh et al., 2010). OCT3/4 acts within a gene regulatory network during early stages of neurodifferentiation (Stefanovic et al., 2007). Shimozaki and others suggest that sustained OCT 3/4 upregulation in Embryonic Stem Cells (ESCs) is required for neurogenesis, and OCT-3/4 with its cofactor SOX2 activates neural transcription factors (Shimozaki et al., 2003). We also observed a substantial but not significant decrease in *Nanog* expression (∼45% decrease, *p* < 0.05, one-way ANOVA). Rodda and others identified *Nanog* as a target for the SOX2 and OCT-3/4 synergistic interaction (Rodda et al., 2005; Pan et al., 2007). Moreover, treatment with a 5:1 PUFA ratio, but not 3:1 ratio significantly decreases expression of the NPC marker genes FOXG1 and *Nestin*. FOX transcription factors play a role in development; however they are also expressed in post-embryonic structures (Hettige et al., 2019). Similar to OCT-3/4 and *Nanog*, Fox family proteins participate in cell cycle regulation, stem cell maintenance and development, cell signalling and control metabolic pathways (Golson et al., 2016; Hettige et al., 2019). FOXG1 is a regulator of neurogenesis, during early cortical cell fate, and plays a role in survival of mature neurons (Zaret et al., 2016; Hettige et al., 2019). Mutations in FOXG1 are causal of a rare neurodevelopmental disorder (FOXG1 syndrome) that results in abnormal brain function. Studies showed that complete loss of *foxg1* in mice is post-natally lethal, while homozygous knockout of the gene in differentiating cells leads to generation of significantly smaller embryoid bodies (Martynoga et al., 2005; Mall et al., 2017; Hettige et al., 2019). It has been shown that during early brain development, FOXG1 can directly control levels of PAX6 and inhibit the premature differentiation of neurons (Manuel et al., 2011; Patriarchi et al., 2016). On the other hand, overexpression of FOXG1 and PAX6 supress gliogenesis and induce neuronal differentiation (Raciti et al., 2013; Patriarchi et al., 2016). The observed decrease in FOXG1 and PAX6 expression that we observe with NPCs treated with high (5:1) ratio of PUFAs suggest that they may negatively affect differentiation and induce faster, premature neurogenesis *in vitro*.

To understand the outcome of these changes, we analysed the effect of different PUFA ratios on mature cortical neurons activity using MEAs. This analysis showed that higher n-3 and intermediate n-6 ratios accelerate neuronal network formation, while high n-6 PUFAs causes a dysfunctional network activity. Balance between excitatory and inhibitory neurotransmission is essential for normal neuronal activity and disruption in E/I signaling is associated with a number of neuropsychiatric and neurodegenerative disorders (Musazzi et al., 2013; Kantamneni, 2015; Tatti et al., 2017; Cherubini et al., 2022). Alsaqati and coworkers performed similar MEA analysis of ASD-patient derived neurons with a functional loss of TSC2 and also found a significantly increased neuronal firing rate compared with controls, but dysfunctional network activity with reduced synchronization. Again, this was due to an E/I imbalance and elevated expression of GABA_A_ receptor genes (Alsaqati et al., 2020). Although not identical, a similar mechanism may generate an E/I imbalance when hiPSC cells are treated with a high n-6 PUFA ratio.

Consistent with this hypothesis, our RNAseq analysis revealed a sustained increase in GABA receptor expression in D20 NPCs treated with high PUFA ratios (3:1 and 5:1) and a significant increase of GABA receptor genes in high n-6 PUFA in D60 neurons. An interesting question is why the intermediate 3:1 n-6 PUFA ratio differs from the higher 5:1 PUFA ratio. A major difference between these two n-6 PUFA ratios is their effect on FOXG1 and Nestin gene expression, which show substantial and significant decrease in the 5:1 PUFA ratio compared to the 3:1 ratio. Previous reports have shown that FOXG1 downregulation may lead to elevated generation of inhibitory synapses. Patriarchi and others studied the effect of FOXG1^+/−^ mutations on glutamatergic neurons differentiation *in vitro* in patient derived iPSCs and they found that they drive neurodevelopment towards inhibitory cell fate to cause an excitation to inhibition (E/I) shift (Patriarchi et al., 2016). Moreover, evidence also indicates that Nestin controls neuronal differentiation and regulates cortical (excitatory neural progenitor formation (Xue et al., 2010; Bernal et al., 2018; Villalba et al., 2020). These data for FOXG1 and *Nestin* expression may explain why only high n-6 PUFA ratio results in an abnormal neuronal network function.

The transition to the Western diet experienced in the urbanised environment is a threat to human mental health with an increased uptake of n-6 PUFAs may enhance risk of inflammatory and developmental disorders (van Elst et al., 2014). By understanding the pathophysiological mechanisms by which n-6 and n-3 PUFAs alter brain development and function, it may not only be possible to improve public health strategy, but also offers alternative approaches for treatment of mental health conditions (Wainberg et al., 2017). Here, we present a human cell-based approach to investigate the effects of PUFAs on neurodevelopment and use it to simulate the effects of an imbalance of the n-6:n-3 PUFA ratios experienced in the diet. This model may fill a gap between basic knowledge about the impact of PUFAs on electrophysiological activity of neurons and inform recommendations in therapy of neuropsychiatric disorders.

In the developing brain, neural stem cell proliferation and subsequent differentiation into neurons and glia cells is regulated by a series of signals. These are important for both the stem cell NPC populations, of which the latter have a limited life span and decreased self-renewal capacity. Cell number, regulated by the balance between pro-death and pro-survival signals, and cell developmental fate are controlled by the activity of transcription factors. In this study we established that high n-6:n-3 PUFA ratio affect the expression of important neuronal transcription factors that control early neurodevelopment and have effects on developed neuronal function. Overall, our data provide a possible molecular mechanism for the impact of n-6 PUFAs on neurogenesis and neuronal activity, where n-6 dietary PUFAs lead to downregulation of the transcription factors FOXG1 and Nestin leading to abnormal neurodifferentiation and an excitatory/inhibitory imbalance of *in vitro* neuronal network. The results of this study, specifically the impact of high PUFA ratio on excitatory/inhibitory imbalance, may have implications for the clinics and the phenotype of patients with neurodevelopmental disorders. A good example is Tuberous Sclerosis Complex, a rare developmental disorder caused by mutations in TSC1 and TSC2. The typical neuro phenotype of TSC patients includes epilepsy, learning disabilities, hyperactivity, and Autism Spectrum Disorder. The multi electrode array analysis of TSC patient derived neurons has shown a similar pattern to what we observed in our control cells treated with high PUFA ratio (5:1) including hyperexcitability and loss of synchronicity in tested neuronal network (Alsaqati et al., 2020). Hence, we speculate that patients with neurodevelopmental disorders such as TSC may by hypersensitive to high PUFA ratios in diet, and changes in dietary habits may have beneficial effects and alleviate symptoms of the disorder.

Although this study focuses on the dietary PUFAs, it does not investigate their immediate effects on n-6 and n-3 PUFA metabolites, LC-PUFAs and oxylipins. These are a diverse set of low molecular weight products, which very often play opposite role in specific molecular pathways regulation. A next step is to understand how changes of the n-6:n-3 PUFA ratio alter the complex profile of their derived signals and related this to the gene expression and developmental changes observed in our cell-based approach. By expanding our metabolomic profile and its integration with transcriptomic and function outputs, we will build a complete understanding of how dietary PUFA exposure leads to altered neurodevelopment.

## Data Availability

The data presented in the study are deposited in the Gene Expression Omnibus repository, accession number GSE228997.

## Abbreviations

ALA, alpha linolenic acid; ARA, arachidonic acid; CTs, clinical trials; DEGs, differentially expressed genes; DHA, docosahexaenoic acid; EFAs, essential fatty acids; EPA, eicosapentaenoic acid; hiPSCs, human induced pluripotent stem cells; LA, linoleic acid; LC-PUFAs, long chain polyunsaturated fatty acids; NPCs, neuronal progenitor cells; PUFA, polyunsaturated fatty acids; SCs, stem cells.
